# Supplementary material for: Desensitization of opsin responses during all-optical interrogation depends on imaging parameters
Source: Neurophotonics. 2026 Jan 24;13(1):015007. doi: 10.1117/1.NPh.13.1.015007 (PMC12831606; doi:10.1117/1.NPh.13.1.015007)
Supplement: Supplementary file 1 [file NPh_013_015007_SD001.pdf]

Supplementary Figures

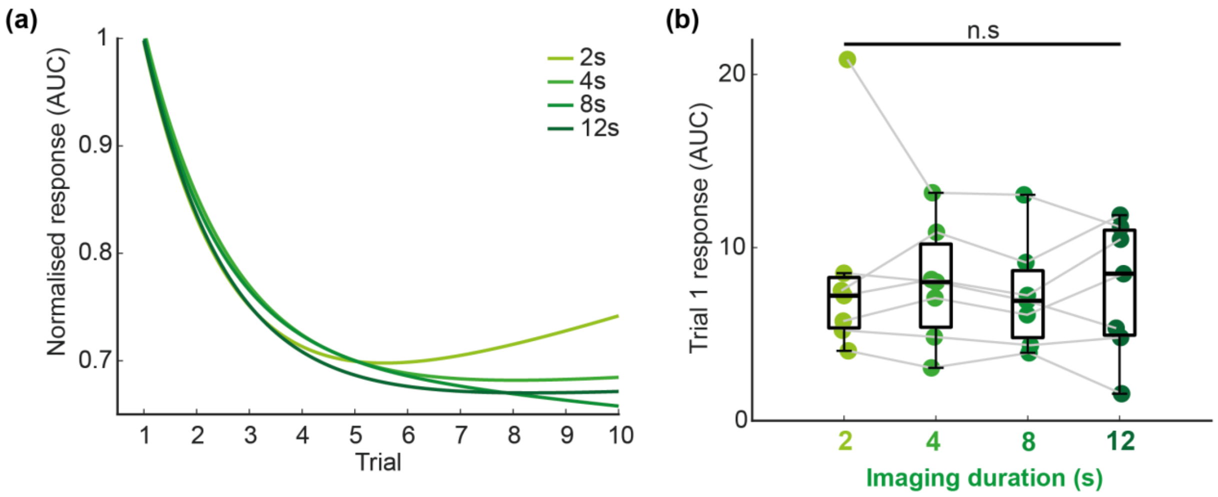

Figure S1 | Targeted photostimulation response dynamics over trials under different imaging conditions

(a) Normalized response (mean trial response across animals / mean trial 1 response across animals) as a function of trial number (n = 7 mice). **(b)** Responses on trial 1 as a function of imaging dose (n.s = p > 0.05, Kruskal-Wallis test, n = 7 mice). Box plots show the median, upper quartile, lower quartile, maximum and minimum, excluding outliers (< lower quartile - 1.5 * inter-quartile range OR > upper quartile + 1.5 * inter-quartile range). Each dot represents an animal. Grey lines connect data from the same animal.

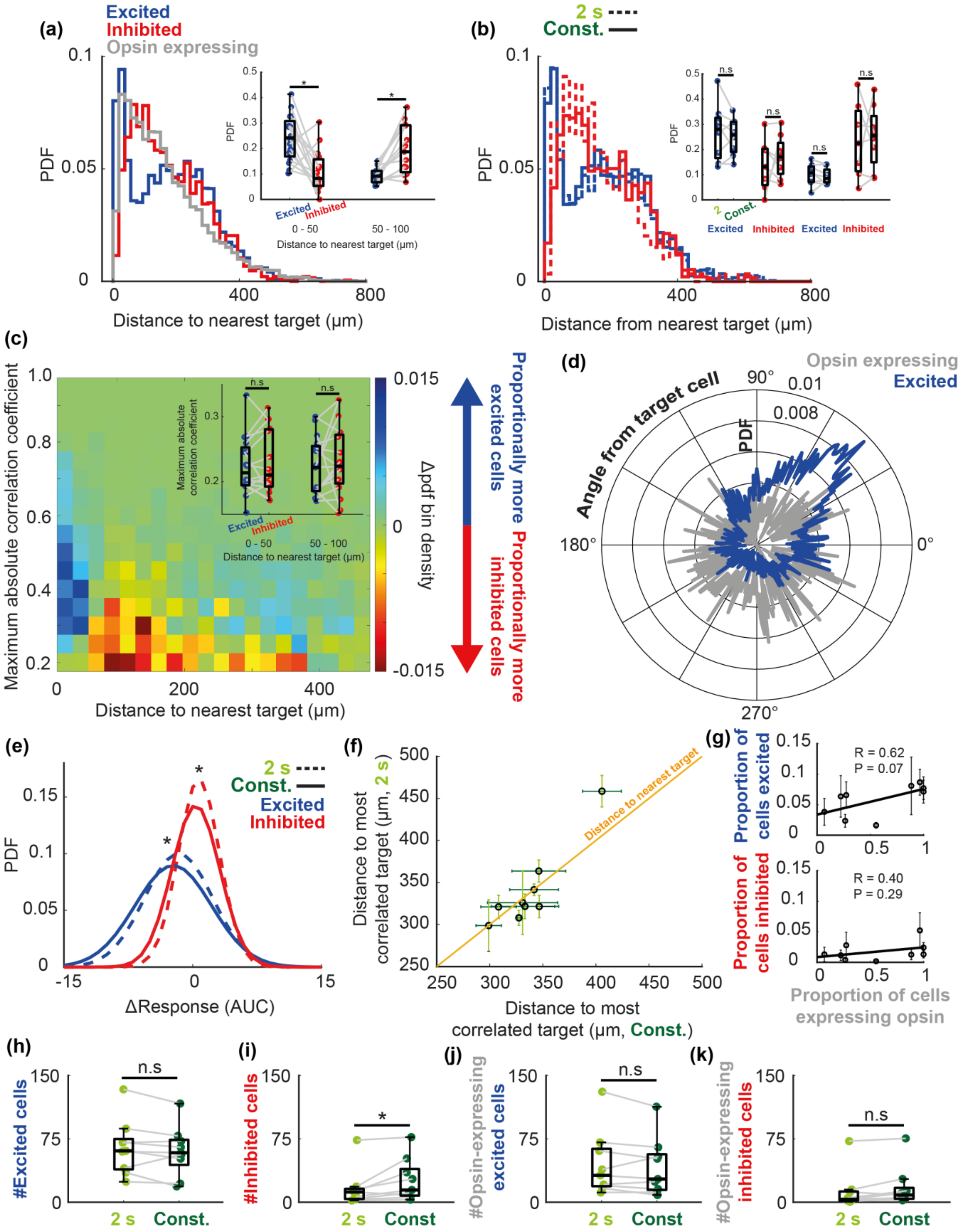

Figure S2 | Spatial distribution of excited and inhibited cells under different imaging conditions **(a)** Probability distribution of excited, inhibited and opsin-expressing non-target cells as a function of distance from the nearest target cell. Inset: Each point is data from an individual animal, averaged

across all cells across all conditions (* = $p < 0.05$, Wilcoxon signed-rank test, n = 16 mice). Box plots show the median, upper quartile, lower quartile, maximum and minimum, excluding outliers (< lower quartile - 1.5 * inter-quartile range OR > upper quartile + 1.5 * inter-quartile range). Grey lines connect data from the same animal. **(b)** same as in **(a)** but separated by imaging condition. Inset: (* = $p < 0.05$, Wilcoxon signed-rank test, n = 9 mice). **(c)** Maximum correlation strength of an individual non-target cell with any target as a function of distance from the nearest target cell, color-coded by excited bin count - inhibited bin count. Inset: Each point is data from an individual animal, averaged across all cells for a given type across all conditions (* = $p < 0.05$, Wilcoxon signed-rank test, n = 16 mice). **(d)** Polar plot of an example mouse with opsin expression confined to a part of the field of view. The polar plot shows the probability distribution for excited and opsin-expressing cells at different angles from a target cell, averaged across targets across all conditions. The probability distributions for excited and opsin-expressing cells are non-overlapping **(e)** probability distribution of excited and inhibited cells under different imaging conditions (* = $p < 0.05$, two-sample Kolmogorov-Smirnov test, pooled across n = 9 mice). **(f)** Mean ±SEM distance of non-target cells from their most correlated target cell under different imaging conditions. Orange line is a representation of what a 1:1 relationship would look like, for example if distance to nearest target was plotted on each axis instead, as this would not vary between imaging conditions. **(g)** Correlation between the proportion of cells in the field of view that are excited/inhibited and the proportion of cells expressing opsin (R = Pearson correlation coefficient). **(h-k)** The number of different classifications of non-target cells under different imaging conditions (* = $p < 0.05$, Wilcoxon signed-rank test, n = 9 mice).

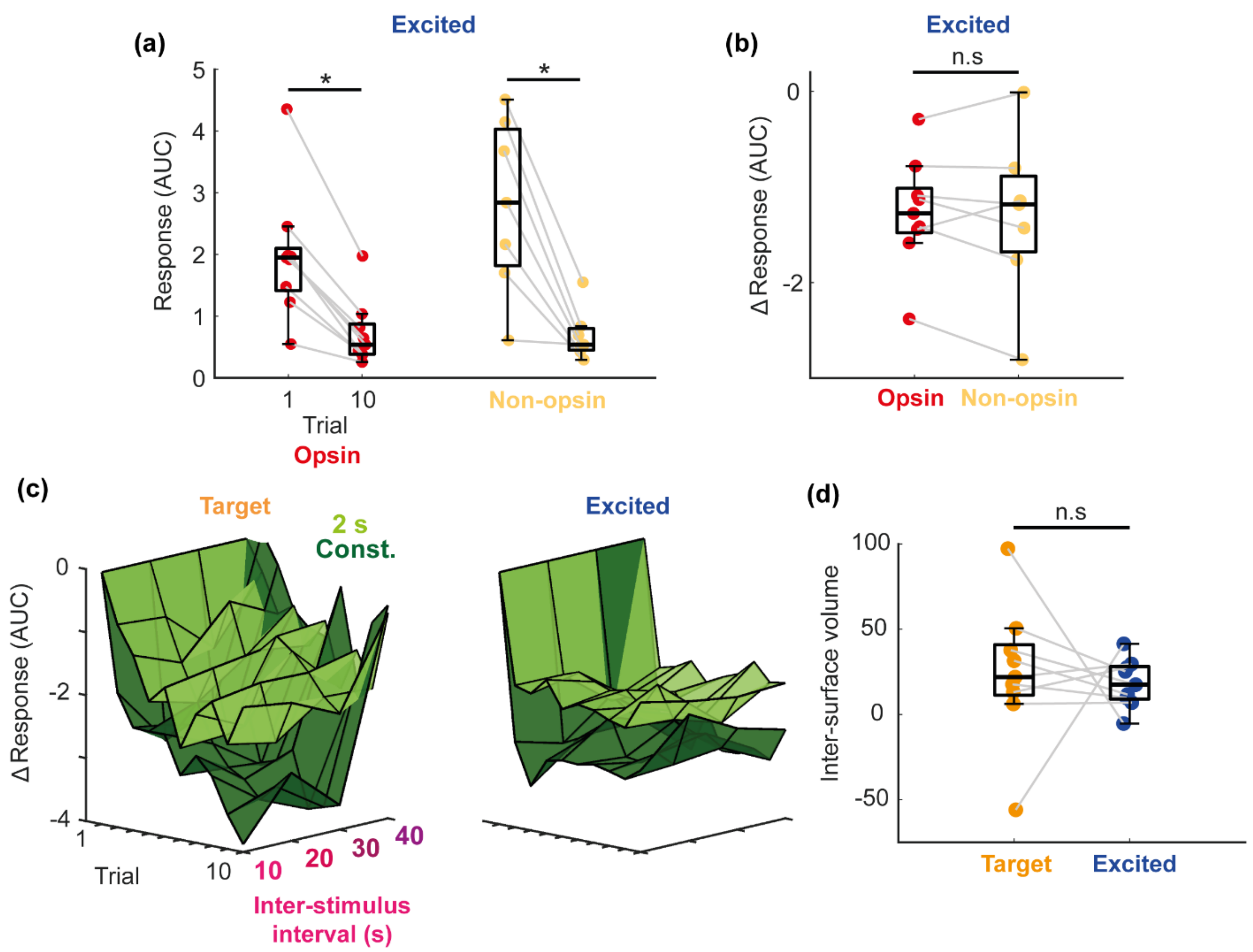

Figure S3 | Desensitization of excited cell responses cannot be rescued by a 40 s inter-stimulus interval

(a) Responses on trials 1 and 10 in opsin-expressing and non-opsin-expressing excited cells, averaged across imaging conditions and inter-stimulus intervals (* = $p < 0.05$, Wilcoxon signed-rank test, n = 9 mice for opsin-expressing excited cells; n = 7 mice for non-opsin-expressing excited cells). Box plots show the median, upper quartile, lower quartile, maximum and minimum, excluding outliers (< lower quartile - 1.5 * inter-quartile range OR > upper quartile + 1.5 * inter-quartile range). Each dot represents an animal. Grey lines connect data from the same animal. **(b)** ΔResponses (trial 10 - trial 1 response) in opsin-expressing and non-opsin-expressing excited cells, averaged across imaging conditions and inter-stimulus intervals (* = $p < 0.05$, Wilcoxon signed-rank test). **(c)** Target cell (left) and excited cell (right) ΔResponses (trial response - trial 1 response) across trials and inter-stimulus intervals under different imaging conditions. **(d)** Volume differences between the two second surface plot and constant imaging surface plot for target and excited cells (area under two-second imaging surface - area under constant imaging surface. * = $p < 0.05$, Wilcoxon signed-rank test, n = 9 mice with somatosensory opsin expression).
